# Unsymmetrical Spiroalkanedithiols Having Mixed Fluorinated and Alkyl Tailgroups of Varying Length: Film Structure and Interfacial Properties

**DOI:** 10.3390/molecules23102632

**Published:** 2018-10-13

**Authors:** Pawilai Chinwangso, Lydia R. St. Hill, Maria D. Marquez, T. Randall Lee

**Affiliations:** Department of Chemistry and the Texas Center for Superconductivity, University of Houston, 4800 Calhoun Road, Houston, TX 77204-5003, USA; pawilaichinwangso@yahoo.com (P.C.); lydiasthill@gmail.com (L.R.S.H.); mdmarquez2@uh.edu (M.D.M.)

**Keywords:** spiroalkanedithiols, homogeneously mixed monolayers, self-assembled monolayers, SAMs, fluorinated

## Abstract

A custom-designed series of unsymmetrical spiroalkanedithiols having tailgroups comprised of a terminally fluorinated chain and a hydrocarbon chain of varying lengths were synthesized and used to prepare self-assembled monolayers (SAMs) on gold substrates. The specific structure of the adsorbates was of the form [CH_3_(CH_2_)_n_][CF_3_(CF_2_)_7_(CH_2_)_8_]C[CH_2_SH]_2_, where n = 7, 9, and 15 (designated as **F8H10-C10**, **F8H10-C12**, and **F8H10-C18**, respectively). The influence of the length of the hydrocarbon chain in the bidentate dithiol on the structure and interfacial properties of the monolayer was explored. A structurally analogous partially fluorinated monodentate alkanethiol and the corresponding normal alkanethiols were used to generate appropriate SAMs as reference systems. Measurements of ellipsometric thickness showed an unexpectedly low film thickness for the SAMs derived from the bidentate adsorbates, possibly due to disruptions in interchain packing caused by the fluorocarbon chains (i.e., phase-incompatible fluorocarbon-hydrocarbon interactions), ultimately giving rise to loosely packed and disordered films. Analysis by X-ray photoelectron spectroscopy (XPS) were also consistent with a model in which the films were loosely packed; additionally, the XPS spectra confirmed the attachment of the sulfur headgroups of the bidentate adsorbates onto the gold substrates. Studies of the SAMs by polarization modulation-infrared reflection-adsorption spectroscopy (PM-IRRAS) suggested that as the length of the hydrocarbon chain in the adsorbates was extended, a more ordered surface was achieved by reducing the tilt of the fluorocarbon segment. The wettability data indicated that the adsorbates with longer alkyl chains were less wettable than those with shorter alkyl chains, likely due to an increase in interchain van der Waals forces in the former.

## 1. Introduction

Over the last three decades, self-assembly has developed into a technique that is widely used to generate well-defined organic thin films on metal substrates [1,2,3,4]. The resultant films, denoted as self-assembled monolayers (SAMs), offer great potential for use in a number of applications, such as corrosion inhibition [5,6,7,8,9], lithographic patterning [10,11], biomaterials fabrication [12,13,14,15,16,17,18], and microelectronic device manufacturing [19,20,21,22]. In particular, alkanethiolate-based monolayers on gold substrates are the most studied system, due to their reproducible ordered structures and relatively simple preparation procedures [2,23]. The interfacial properties of the organic surfaces, such as wettability [24,25], adhesion [26,27,28,29], and friction [30,31,32,33,34,35,36], can be controlled by varying the structure of the adsorbate employed.

A major focus of our research has centered on the development of specifically fluorinated thin films with varying degrees of fluorination [37,38,39,40]. Fluorocarbon-based films offer many advantages over hydrocarbon-based films, including thermal stability, chemical inertness, hydrophobicity, and oleophobicity [37,38,39,40,41,42,43]. However, these properties are greatly influenced by the degree of fluorination on the molecular constituents. Of particular interest are the contact angle studies performed on SAMs generated from specifically fluorinated alkanethiols [41,42,43,44,45,46,47,48]. Table 1 highlights studies of the wetting properties of partially fluorinated SAMs. In one example of minimally fluorinated SAMs, Lee et al. demonstrated that CF_3_-termianted SAMs exhibit greater wettability toward polar contacting liquids than their hydrocarbon analogs [43,45,46,47]. The enhanced wettability exhibited by the CF_3_-terminated SAMs was attributed to the presence of oriented dipoles at the fluorocarbon-hydrocarbon junction, FC-HC. Further research examined the surface free energy as a function of the degree of terminal fluorination by analyzing SAMs generated from two series of adsorbates in which: (1) The total carbon count remains the same while the number of fluorocarbons systematically increases, F_3_C(CF_2_)_n_(CH_2_)_m_SH where n = 0–9 and m = 15–6 [46]; and (2) the hydrocarbon spacer remains the same while the number of fluorocarbons increases F_3_C(CF_2_)_n_(CH_2_)_m_SH where n = 0–9 and m = 11 [39,48]. Collectively, these studies led to the conclusions that the contact angles are affected by the number of fluorocarbons rather than the total chain length, and that the films become increasingly hydrophobic and oleophobic as the degree of fluorination increases. A more in-depth discussion on the influence fluorination has on the structural and interfacial properties of SAMs can be found in the literature [40,44].

Much of the previously mentioned research has been obtained from ω-terminated alkanethiols (monothiol-based adsorbates), which provide a single point of contact between the sulfur headgroup and the metal surface. Alternatively, other types of adsorbates having multiple headgroup-metal interaction points have also been developed and studied [49,50,51]. Bidentate adsorbates have been introduced into surface chemistry, due to their ability to enhance the stability of thin films formed on solid substrates [52]. The enhanced stability exhibited in films derived from multidentate adsorbates has been attributed to an entropy-driven phenomenon known as the “chelate effect”, which is a key strategy used to enhance the stability of metal-ligand complexes in inorganic materials [53,54,55,56]. Partial desorption of a bidentate adsorbate leads to a situation where one arm of the adsorbate is bound to the surface while the other is desorbed, but held nearby covalently; in an entropic sense, the desorbed arm is more likely to bind again to the surface than the case for a monodentate adsorbate, where the desorbed molecule can readily diffuse away. This concept can be readily applied to films derived from organic molecules covalently bound to surfaces by incorporating multiple attachment points on the adsorbate to create multiple interactions between the adsorbate and the metal surface. Several examples of designs of such bidentate adsorbates, which have been used to enhance film stability on metal substrates, include aromatic-based bidentate adsorbates [56,57,58,59], spiroalkanedithiolate-based adsorbates [60,61,62,63], and trithiolate-based adsorbates [64,65,66,67,68,69]. Using the spiroalkanedithiol (SADT) structure, well-packed and highly oriented SAMs can be generated by utilizing an adsorbate structure having a pair of relatively long alkyl chains [60]. A major attractive feature of this design is the relatively simple synthetic approach, which can open new avenues for the synthesis of a large variety of adsorbates. A recent example includes the development of film compositions where two phase-incompatible groups were incorporated into the same molecule, yielding homogeneously mixed SAMs [62,63]. Important to note are previous efforts toward the development of mixed-chained adsorbates originating from a single point, including the use of dialkyl sulfides by Whitesides [70], as well as other methods highlighted in the literature [51]. However, SAMs generated from dialkyl sulfides, asymmetrical disulfides, and other methods described in the literature used to generate mixed SAMs suffer from poor stability, disordered films, and/or preferential adsorption, which can be circumvented when the SADT structure is incorporated into the architecture of the adsorbate [25,62,63].

**Figure 1:**
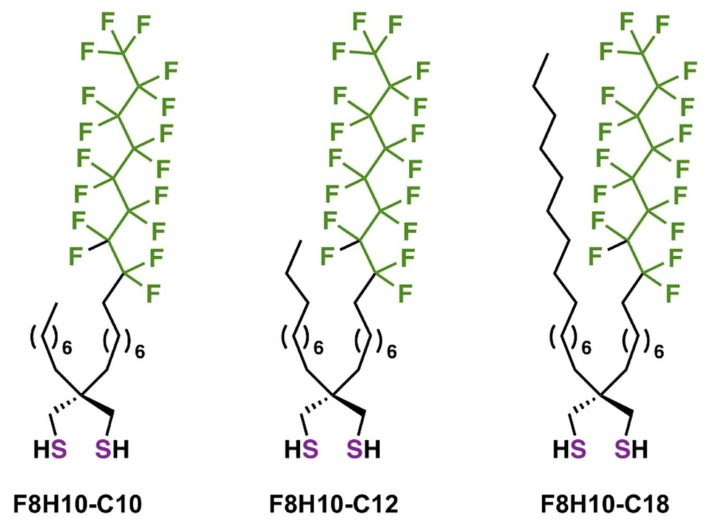
Molecular structures of the unsymmetrical partially fluorinated spiroalkanedithiols (**F8H10-Cm**, m = 10, 12, and 18) examined in the present study: **F8H10-C10**, **F8H10-C12**, and **F8H10-C18**.

In a previous report, we studied the synthesis and characterization of SAMs generated from a prototype unsymmetrical partially fluorinated SADT [62]. The structure consisted of the spiro-dithiol headgroup connected with two different tailgroups (i.e., one hydrocarbon and one partially fluorinated), as shown in Figure 1. This specific design was motivated by the potential development of thin film fluorinated surfaces to mimic poly(tetrafluoroethylene). The discovery of poly(tetrafluoroethylene) (PTFE), by Plunkett in 1938 [71], has led to the extensive use of fluoropolymers in various applications, due to their chemical inertness, thermal and mechanical resistance, and low adhesion [72]. Therefore, fluorocarbon-based films can offer certain advantages over hydrocarbon-based films in terms of chemical resistance, chain rigidity, thermal stability, hydrophobicity, and oleophobicity [37,38,41]. For this report, two new fluorinated bidentate dithiols were synthesized having the molecular structures shown in Figure 1.

**Figure 2:**
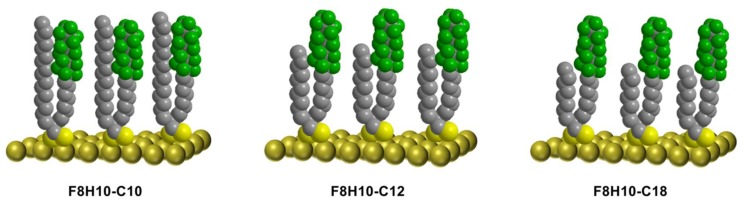
Illustration of idealized SAMs on gold derived from **F8H10-C10**, **F8H10-C12**, and **F8H10-C18**.

Notably, the original prototype in Figure 1, [CH_3_(CH_2_)_7_][CF_3_(CF_2_)_7_(CH_2_)_8_]C[CH_2_SH]_2_ (**F8H10-C10**), possesses a partially fluorinated chain and a shorter hydrocarbon chain having the same number of hydrocarbon units as that in the partially fluorinated chain. This original adsorbate was designed to form SAMs, in which the helical fluorinated chains (van der Waals diameter of ~5.6 Å) [73] would not be subjected to the packing constraints typically observed in SAMs derived from the corresponding monothiol, but instead pack on top of the underlying well-packed trans-extended alkyl chains (van der Waals diameter of ~4.2 Å) [74]. To explore further the effects of molecular structure on the packing density of the chains and interfacial properties of SAMs derived from this class of adsorbate, the present study examines SAMs derived from the partially fluorinated spiroalkanedithiols in Figure 1 (i.e., **F8H10-C12** and **F8H10-C18**, respectively). In efforts to examine the effects of hydrocarbon chain length on film properties, we incorporated systematically longer hydrocarbon chains in these two new adsorbate molecules. A schematic representation illustrating a well-organized SAM for each of the studied adsorbates is shown in Figure 2. 

Further, we compared the **F8H10-Cm** SAMs to SAMs derived from the corresponding monodentate n-alkanethiols: CH_3_(CH_2_)_n_SH, where n = 9, 11, and 17 (**C10SH**, **C12SH**, and **C18SH**, respectively) and the structurally analogous partially fluorinated monodentate thiol CF_3_(CF_2_)_7_(CH_2_)_10_SH (**F8H10SH**). We characterized the structure and the interfacial properties of all SAMs using ellipsometry, X-ray photoelectron spectroscopy (XPS), polarization modulation infrared reflection-adsorption spectroscopy (PM-IRRAS), and contact angle goniometry. 

## 2. Results and Discussion

The **F8H10-Cm** SAMs were allowed to equilibrate at room temperature for 48 h in DMF prior to characterization. Previous work on related SADT SAMs developed in DMF yielded films with optimal qualities (i.e., relatively ordered, and less wettable, with a well-packed monolayer film) [62]. We also prepared SAMs from the corresponding monodentate thiols to serve as reference systems. For instance, we assumed that the data from SAMs derived from **F8H10-C10** could be readily compared to those derived from **C10SH** and **F8H10SH**. This assumption allows a direct evaluation of the SADT SAMs against SAMs of analogous structural character. Consequently, we prepared SAMs derived from the normal alkanethiols **C10SH**, **C12SH**, and **C18SH** along with the structurally analogous fluorinated **F8H10SH** for comparison with the SAMs derived from **F8H10-C10**, **F8H10-C12**, and **F8H10-C18**, respectively.

### 2.1. Film Thicknesses

Preliminary confirmation of SAM formation on metal substrates can be determined by measuring the thickness of the film using ellipsometry [62]. Table 2 shows the thicknesses obtained for all of the SAMs. The SAMs derived from the monodentate thiols gave values that correspond to those reported in the literature, 10, 12, 22, and 18 Å for the **C10SH**, **C12SH**, **C18SH**, and **F8H10SH** SAMs, respectively [42,75,76].

Comparison of the thickness values obtained for the monodentate SAMs and the **F8H10-Cm** SAMs indicates that the **F8H10-Cm** SAMs exhibit thickness values that are consistent with the formation of monolayer films. Furthermore, as the alkyl chain length is increased systematically in the series, **F8H10-C10**, **F8H10-C12**, and **F8H10-C18**, the thicknesses increase systematically as well: 14 Å, 16 Å, and 21 Å, respectively. The observation that the thickness of the **F8H10-C18** SAM is comparable to that of the **C18SH** SAM (i.e., 21 Å verses 22 Å, respectively) suggests that the chains in the **F8H10-C18** SAM are densely packed. Furthermore, the lower thickness values observed for the **F8H10-C10** and **F8H10-C12** SAMs compared to that for the **F8H10SH** SAM (18 Å) are consistent with a model, in which the alkyl chains in the bidentate films underlay a fluorocarbon chain that is loosely packed with room to undergo significant chain tilt, in order to maximize interchain van der Waals interactions with neighboring fluorocarbon moieties [62].

### 2.2. Analysis of the Films by XPS

**Figure 3:**
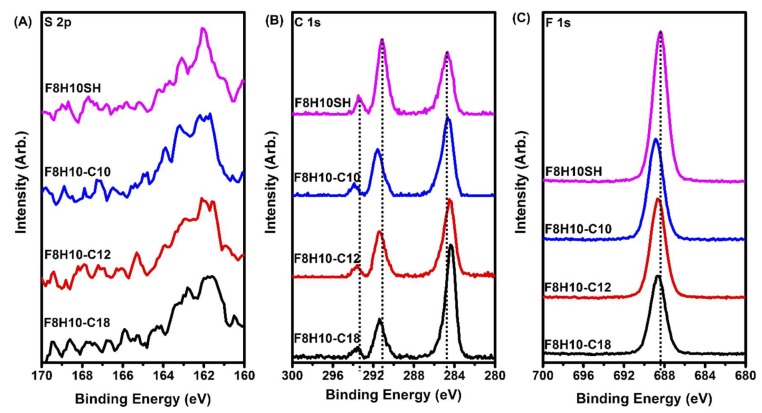
X-ray photoelectron spectroscopy (XPS) spectra collected from SAMs derived from **F8H10SH** and **F8H10-Cm** (m = 10, 12, and 18) for the (**A**) S 2p; (**B**) C 1s; and (**C**) F 1s regions.

Figure 3 provides the XPS spectra of the S 2p, C 1s, and F 1s regions for the SAMs derived from **F8H10SH** and the **F8H10-Cm** adsorbates. Figure 3A shows that the S 2p region is comprised of a doublet corresponding to the S 2p_3/2_ and S 2p_1/2_ photoelectrons. Previous research on SAMs derived from *n*-alkanethiols on gold has shown that the S 2p_3/2_ peak with a binding energy of ~162.0 eV is representative of a bound thiolate, while the S 2p_3/2_ of an unbound sulfur or a disulfide appears at ~164.0 eV [77]. The same observation has also been made for SAMs derived from partially fluorinated alkanethiols adsorbed on gold substrates [62,73]. For the **F8H10SH** SAM and the **F8H10-Cm** SAMs, the observed S 2p_3/2_ peak position suggests that all sulfur moieties in the films are bound to the gold surface.

The C1s spectra for the **F8H10SH** and the SADTs are shown in Figure 3B, and reveal three peaks characteristic of CH_2_, CF_2_, and CF_3_ units at ~285 eV, 292 eV, and 294 eV, respectively [42,73]. Comparison of the C1s (CH_2_) peak positions of the **F8H10-Cm** SAMs to those of the **F8H10SH** shows a systematic decrease in the binding energy as the length of the hydrocarbon chain increases. The densely packed SAMs derived from **F8H10SH** and those from **F8H10-C10** exhibit a binding energy of 284.7 eV, while the SAMs derived from **F8H10-C12** and **F8H10-C18** exhibit values of 284.5 eV and 284.4 eV, respectively. The lower binding energies of the **F8H10-C12** and **F8H10-C18** SAMs compared to the **F8H10SH** and **F8H10-C10** SAMs are indicative of a lower hydrocarbon chain packing density in the former films [78]. Furthermore, the shift to lower binding energies in the **F8H10-C12** and **F8H10-C18** SAMs as the length of the hydrocarbon chain is increased can be plausibly attributed to phase-incompatible fluorocarbon-hydrocarbon interactions in these films. Overall, the relative alkyl chain density of the fluorinated adsorbates decreases as follows: **F8H10SH**~**F8H10-C10** > **F8H10-C12** > **F8H10-C18**.

**Figure 4:**
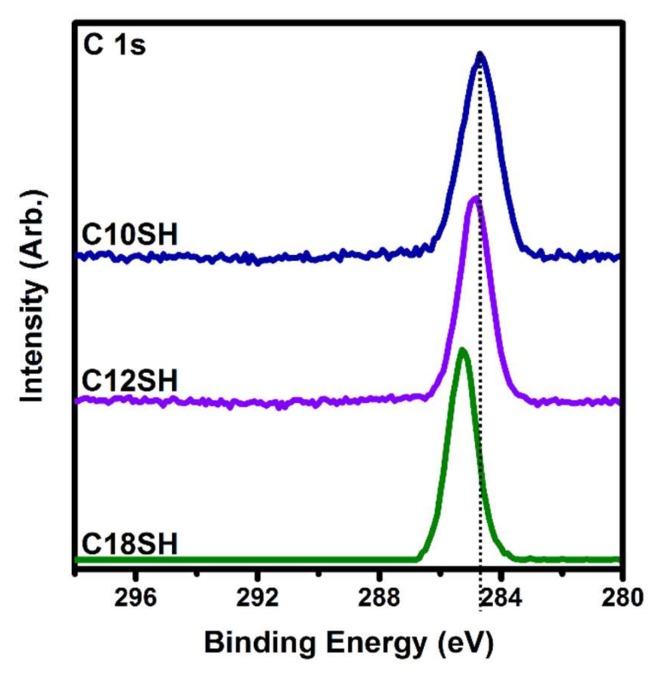
XPS spectra of the C 1s region collected from SAMs derived from the normal alkanethiols, **C10SH**, **C12SH**, and **C18SH**.

Several studies on SAMs derived from *n*-alkanethiols have shown that the position of the C 1s peak can be used as a rough measurement of surface coverage for the adsorbates on the substrate [42,73,79,80,81]. In a series of *n*-alkanethiols with successively increasing chain length, the binding energy of the C 1s photoemission shifts to a higher value when the alkyl chain length is increased, which is attributable to an increase in the packing density of the alkyl chains, as well as a film with minimal gauche defects [79,80,82,83]. As shown in Figure 4 and Table 3, we observe the same trend for SAMs derived from *n*-alkanethiols on gold: The C 1s (CH_2_) peak shifts from 284.7 eV for **C10SH** to 284.8 eV for **C12SH** to 285.3 eV for **C18SH**. The higher binding energy of the densely packed alkanethiolate monolayers have an increased resistance to photoelectron emission from the surface during the irradiation process, shifting the peak position to a higher binding energy [79,80,82]. 

Considering the peak positions of the fluorinated components (CF_2_, CF_3_, and F peaks) in the XPS spectra—Figure 3B,C, and Table 3—the C 1s (CF_2_ and CF_3_) and F1s peaks shift to higher binding energies as compared to those of the **F8H10SH** film in the following order: **F8H10SH** < **F8H10-C18** ≤ **F8H10-C12** < **F8H10-C10**, which can be ascribed to the structural parameters of each adsorbate on the substrate. Frey et al. and Tamada et al. also observed these shifts to higher binding energies of the fluorinated components in their studies of partially fluorinated alkanethiol SAMs when the length of the underlying hydrocarbon chain was increased [42,73]. The authors rationalized this phenomenon by relating a screening effect for the core excited atoms in conjunction with the distance between the core hole and the substrate. The surface charge generated from the photoelectron emission of a core carbon or a fluorine atom in the fluorocarbon segment during X-ray irradiation can be discharged by the electrons in the substrate, which can move to screen the core hole within the film [84]. If the distance between the core hole and the substrate increases, the monolayers may not be discharged completely; therefore, an increase in thickness of the hydrocarbon segment below the fluorocarbon layer was responsible for the shift to higher binding energies of the C 1s (CF_2_), C 1s (CF_3_), and F 1s peaks [73]. 

Comparison of the C 1s peaks for the CF_2_, CF_3_, and the F 1s peaks for the SAMs derived from **F8H10SH** and the bidentate SADTs shows that the **F8H10SH** SAM exhibits the lowest binding energies (~291.2 eV, 293.5 eV, and 688.4 eV), while the **F8H10-C10** SAM exhibits the highest binding energies (~291.6 eV, 293.9 eV, and 688.9 eV), with the **F8H10-C18** and **F8H10-C12** SAMs in between (~291.4 eV, 293.6 eV and 688.7 eV). While it is tempting to try to draw conclusions regarding the chain packing in the hydrocarbon segments from these data, we recognize that the electronic environment of the fluorocarbon chains might vary greatly in these films as the fluorocarbon chains are densely packed for the **F8H10SH** SAM, loosely packed for the **F8H10-C10** SAM, and probably somewhere in between for the **F8H10-C18** and **F8H10-C12** SAMs. Further insight regarding the orientation of the hydrocarbon and fluorocarbon chains in these films can be obtained from the interpretation of the PM-IRRAS data, which are discussed in a later section [38].

Another aspect of the XPS data that can be analyzed to obtain quantitative results is the intensity of the peak, specifically to reveal the adsorbate density and composition on the substrate [73,85]. Table 4 shows the normalized intensities for the C 1s (CH_2_), C 1s (CF_2_ + CF_3_), and F 1s peaks of each fluorinated adsorbate used in this study; for simplicity, the intensities were normalized with respect to the C 1s (CF_2_ + CF_3_) and the F 1s peak intensities, respectively, of the **F8H10SH** SAM. The peak intensity increase observed for the CH_2_ component of the bidentate dithiols relative to the **F8H10SH** adsorbate is affected by the addition of the second hydrocarbon chain (i.e., an increased number of CH_2_ units) for each of the respective adsorbates. The C 1s (CF_2_ + CF_3_) and F 1s peak intensities for the **F8H10-Cm** SAMs are weaker than those of the **F8H10SH** SAM, which can be attributed to the packing density of the fluorocarbon chains of the SAMs (i.e., **F8H10SH** > **F8H10-Cm**) [73]. The diminished intensity and/or packing density of the chains can also be attributed to the steric bulk and consequently greater space occupied by the branched hydrocarbon chains of the bidentate adsorbates, which can plausibly lead to diminished fluorocarbon density at the interface. 

Among the **F8H10-Cm** SAMs, the relative packing density of the fluorocarbon chain decreases as the hydrocarbon chain length increases, as judged by the F 1s peak intensities, shown in Figure 3 and Table 4, in the following order: **F8H10-C10** ≥ **F8H10-C12** > **F8H10-C18**. It should be noted that the addition of the two methylene units along the hydrocarbon chain for **F8H10-C12** might disturb the fluorocarbon chain order and/or packing. However, the effect appears to be minimal with regard to changes in overall structure and interfacial properties (i.e., thickness, packing density of the chains, and wettability, which will be described subsequently), between the **F8H10-C12** and **F8H10-C10** SAMs. The structure of the **F8H10-C18** film, on the other hand, is influenced by phase-incompatible fluorocarbon-hydrocarbon interactions in the terminal portions of this SAM, which contributes to chain disorder, and thus leads to a more loosely packed SAM.

### 2.3. PM-IRRAS Analysis of the Films

**Figure 5:**
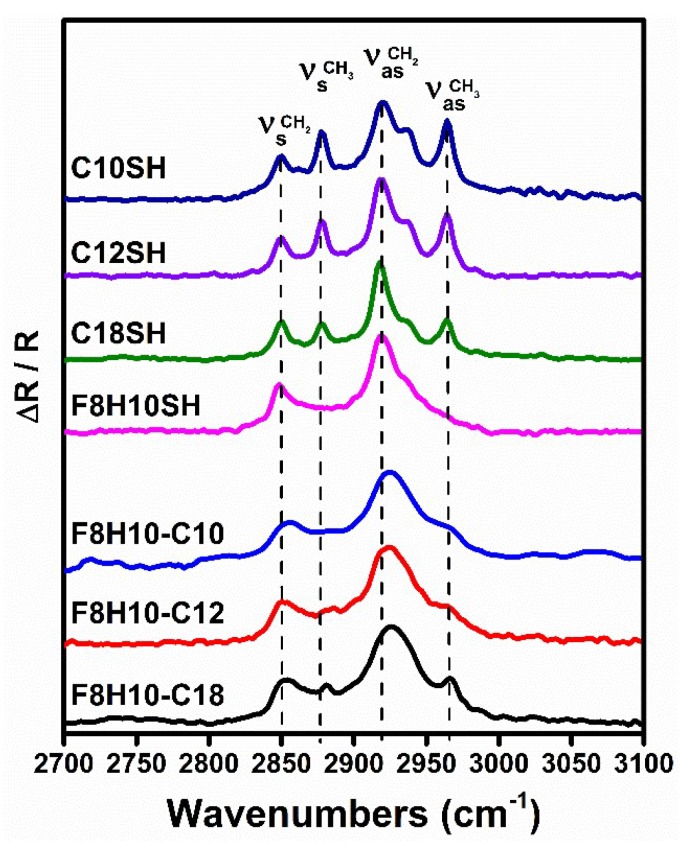
Polarization modulation-infrared reflection-adsorption spectroscopy (PM-IRRAS) spectra in the C-H stretching region for SAMs generated from the alkanethiols, **F8H10SH**, and the **F8H10-Cm** adsorbates used in this study.

**Figure 6:**
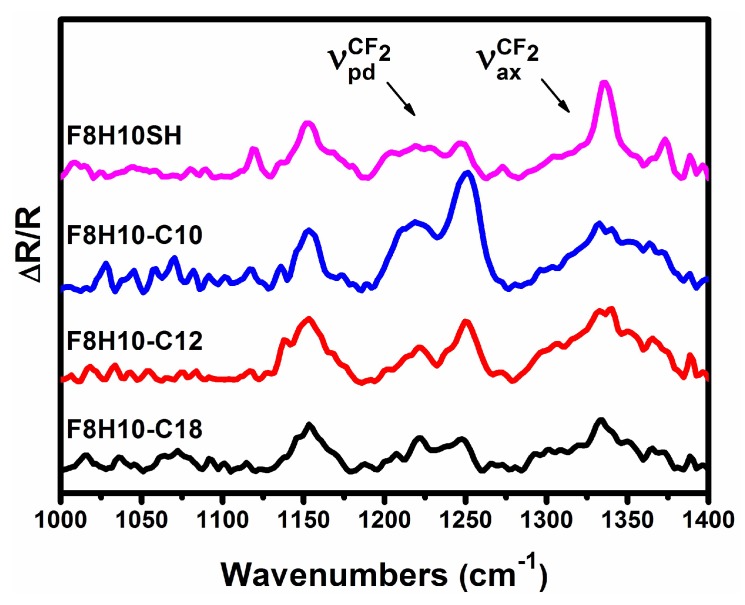
PM-IRRAS spectra of the C-F stretching region for SAMs generated from the **F8H10SH** and **F8H10-Cm** adsorbates used in the study.

The PM-IRRAS spectra for the C-H and C-F stretching regions are depicted in Figure 5 and Figure 6, respectively. The C-H stretching modes are known to be sensitive to the conformational order (i.e., crystallinity) of the hydrocarbon chains [86], while the C-F vibrations can provide information regarding the orientation of the fluorinated segments in fluorocarbon films [38]. The position of the antisymmetric (ν_as_^CH2^) methylene C-H stretching bands has been used as a means to determine the conformational order of a SAM [86]. For a well-ordered (crystalline) film, the ν_as_^CH2^ peak appears at 2918 cm^−1^; however, the ν_as_^CH2^ peak for a disordered (liquid like) film appears at 2924 cm^−1^ [75]. For the SAMs derived from the *n*-alkanethiols, the ν_as_^CH2^ band positions were observed at 2920, 2919, and 2918 cm^−1^ for the **C10SH**, **C12SH**, and **C18SH** SAMs, respectively (see Figure 5). Consistent with the literature data, the degree of conformational order in these SAMs increased with an increase in alkyl chain length [75]. Also apparent in the spectra is the C-H vibrations originating from the methyl groups at 2965 cm^−1^ for the ν_as_^CH3^ band and 2878 cm^−1^ for the ν_s_^CH3^ band. 

For the fluorinated adsorbates, the ν_as_^CH2^ of the **F8H10SH** SAMs appears at 2919 cm^−1^, consistent with a conformationally ordered film. However, the SAMs derived from the SADTs showed a significant shift of the ν_as_^CH2^ band to higher wavenumbers: 2923 cm^−1^ for **F8H10-C10**, 2924 cm^−1^ for **F8H10-C12**, and 2925 cm^−1^ for **F8H10-C18**. The higher wavenumber suggests that the hydrocarbon chains in the bidentate SAMs are less conformationally ordered than those in the monodentate SAMs. This observation can be attributed to a reduction in the chain packing density, due to the branched nature of the adsorbates. The presence of hydrocarbon chains of identical length below the fluorocarbon segment of the **F8H10-C10** SAMs perhaps, allows the alkyl chains to be more densely packed relative to those in the **F8H10-C12** SAM and especially compared to the **F8H10-C18** SAM, where the phase-incompatible fluorocarbon-hydrocarbon interactions disrupt the monolayer packing, which is consistent with the XPS data (vide supra). Another aspect of the spectra to note is the absence of the C-H stretches associated with the methyl group for the **F8H10-C10** and **F8H10-C12** SAMs. The absence of those peaks can be attributed to their location underneath the fluorocarbon portion, a situation that appears to limit the methyl vibrations to coincide with the surface selection rules (i.e., only vibrations causing changes in the transition dipole moment perpendicular to the surface can be detected) [87], leaving these vibrations undetected by the PM-IRRAS measurement.

The bands arising from the C-F vibrations in the region from 1000 to 1400 cm^−1^ (shown in Figure 6 and discussed below), were assigned based on literature examples using similar fluorocarbon materials [37,73]. Additionally, an estimate of the orientation of the fluorocarbon segments can be obtained by comparing the relative intensities of bands associated with transitions perpendicular and parallel to the fluorinated helix, ν_pd_^CF2^ and ν_ax_^CF2^ respectively (vide infra) [73,88,89]. The band at 1150 cm^−1^ has been assigned as the symmetric C-F stretch of the CF_2_, while those at 1205 and 1250 cm^−1^ to the antisymmetric C-F stretches [37,90]. Complicating our analysis of the C-F spectra is the overlap of other vibrational modes, including C-C bond stretching and C-C-C bending at ~1220 cm^−1^ [37,91]. The bands in the 1150–1270 cm^−1^ range can be attributed to the stretches whose transitions dipole moments are perpendicular to the fluorocarbon chain, ν_pd_^CF2^ [38]; correspondingly, the bands at 1336 and 1373 cm^−1^ have been identified as stretches with a transition dipole moment parallel to the fluorocarbon helical axis, ν_ax_^CF2^ [38]. When the fluorocarbon chains are oriented perpendicular to the surface (i.e., less tilted from the surface normal), the intensity of the ν_ax_^CF2^ bands are enhanced relative to that of the ν_pd_^CF2^ bands. On the other hand, when the fluorocarbon chains are oriented along the surface (i.e., more tilted from the surface normal), the intensity of the ν_ax_^CF2^ bands are diminished relative to that of the ν_pd_^CF2^ bands.

Based on this analysis, the higher intensity of the ν_ax_^CF^^2^ band relative to the ν_pd_^CF^^2^ band observed for the **F8H10SH** SAM leads to the conclusion that the fluorocarbon helix is oriented nearly perpendicular to the surface. In contrast, the film generated from **F8H10-C10** shows a strong reduction in the relative intensities of ν_ax_^CF^^2^ bands when compared to the ν_pd_^CF^^2^ bands in its spectra, suggesting a highly tilted fluorocarbon chain. The relative intensities of the ν_ax_^CF^^2^ and ν_pd_^CF^^2^ bands for the SAMs derived from **F8H10-C12** and **F8H10-C18** show similar intensities in their respective bands, indicating a fluorocarbon segment with an intermediate degree of tilt. These observations suggest that the latter bidentate SADT SAMs can support the neighboring fluorocarbon chains in a more upward orientation than is found in the **F8H10-C10** SAM.

### 2.4. Wettability of the Films

**Figure 7:**
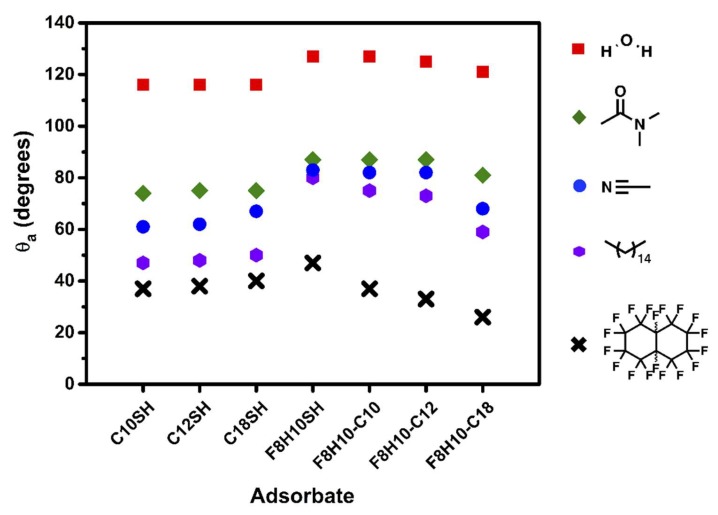
Advancing contact angle data obtained for water (H_2_O), *N*,*N*-dimethylformamide (DMF), acetonitrile (ACN), hexadecane (HD), and perfluorodecalin (PFD) on SAMs derived from the monodentate thiols (**C10SH**, **C12SH**, **C18SH**, and **F8H10SH**) and the **F8H10-Cm** dithiols (**F8H10-C10**, **F8H10-C12**, and **F8H10-C18**).

Contact angle measurements are convenient tools for evaluating the wetting properties of organic interfaces. In this study, we measured advancing contact angles (*θ*_a_) for all SAMs using several probe liquids, ranging from nonpolar (hexadecane (HD, γ_LV_ = 27.5 dynes/cm) and perfluorodecalin (PFD, γ_LV_ = 19.2 dynes/cm)) [92,93], polar aprotic (*N*,*N*-dimethylformamide (DMF, γ_LV_ = 37.1 dynes/cm) and acetonitrile (ACN, γ_LV_ = 29.3 dynes/cm)) [92,93], to polar protic (water (H_2_O, γ_LV_ = 72.8 dynes/cm)) [92,93], as shown in Figure 7 and listed in Table 5. 

**Figure 8:**
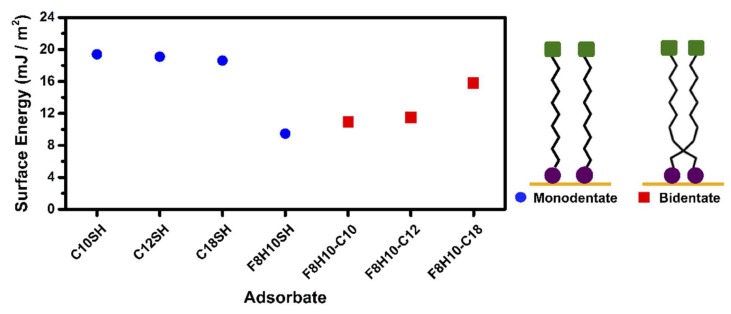
Surface energy calculated using the Owens-Wendt approach for the monodentate thiols and the **F8H10-Cm** dithiols.

The contact angle values obtained from the SAMs derived from the *n*-alkanethiols (**C10SH**, **C12SH**, and **C18SH**) are similar for each of the probe liquids used. The SAMs generated from the short-chained adsorbates, **C10SH** and **C12SH**, proved to be slightly more wettable (lower *θ*_a_) by the liquids having low surface tensions—acetonitrile (29.3 dynes/cm), hexadecane (27.5 dynes/cm) and perfluorodecalin (19.2 dynes/cm) [92,93]—than the SAM generated from the long-chained adsorbate, **C18SH**. The observed differences in the wettability of the *n*-alkanethiol SAMs by the liquids having low surface tension is in accordance with the conformational order and packing of the alkyl chains of the adsorbed molecules, as well as their surface energy. Notably, we used the Owens-Wendt approach to calculate the surface energy of the SAMs (equations and calculations can be found in the Supplementary Information), shown in Figure 8 and Table S2.

The interchain van der Waals forces in a monolayer increase with the addition of more methylene units. The less densely packed SAMs formed from the short-chained adsorbates, **C10SH** and **C12SH**, are more wettable than the more densely packed SAMs derived from the longer-chained adsorbate, **C18SH**. In the SAMs derived from the shorter-chained adsorbates, there is greater atomic contact (dispersive interactions) between the contacting liquid and the SAM interface of the loosely packed SAMs, which leads to a more wettable film [25]. The conformational order and packing density of the films is further reflected in their calculated surface energy, where **C10SH** (19.4 mJ/m^2^) < **C12SH** (19.1 mJ/m^2^) < **C18SH** (18.6 mJ/m^2^). For the SAMs derived from the **F8H10SH**, the contact angle values are higher than those obtained from the **C18SH** film, which is consistent with previously observed values on SAMs derived from partially fluorinated adsorbates with the same degree of terminal fluorination [39,91]. The low surface energy enjoyed by fluorinated interfaces, 9.47 mJ/m^2^ for **F8H10SH**, compared to hydrocarbon interfaces is consistent with the higher contact angle values observed on the **F8H10SH** SAM [40,44,94]. 

For the **F8H10-Cm** SAMs, the contact angle values and their surface energy fall between those of the SAMs derived from their normal alkanethiol and partially fluorinated alkanethiol counterparts, which further suggests the interfacial composition is a mixture of the hydrocarbon and fluorocarbon species in these SAMs; the calculated surface energy of the **F8H10-Cm** series increases in the following order: **F8H10-C10** (10.9 mJ/m^2^) < **F8H10-C12** (11.5 mJ/m^2^) < **F8H10-C18** (15.8 mJ/m^2^). However, perfluorodecalin shows lower contact angle values for the **F8H10-C12** and **F8H10-C18** SAMs when compared to the hydrocarbon and fluorinated analogs. A preliminary analysis might propose that the lower contact angle observed for PFD on these bidentate SAMs might be due to greater attractive interactions (dispersive interactions) between the fluorinated surface and the fluorinated liquid; however, this observation does not hold true for the **F8H10SH** SAM. Therefore, the enhancement in the wettability of the **F8H10-Cm** SAMs is likely due to the low surface tension of perfluorodecalin and the lower chain packing density of the films. The combination of both effects allows for greater dispersive interactions between the chemically similar fluorocarbon surfaces and liquid, facilitating the intercalation of the liquid molecules into the films. 

Figure 7 and Table 5 show that the SAMs derived from the **F8H10-C10**, **F8H10-C12**, and **F8H10SH** adsorbates exhibit similar wettability when in contact with polar contacting liquids, although the **F8H10-C18** SAM was slightly more wettable toward these probe liquids. The lower contact angle values for the **F8H10-C18** SAM likely arise from the diminished chain packing density of this film, vide supra. The decreased chain packing density of the **F8H10-C18** SAM allows for greater intercalation into the film by small molecules—a trend illustrated by the contact angle values of ACN: 83°, 82°, 82°, and 68° for the series **F8H10SH**, **F8H10-C10**, **F8H10-C12**, and **F8H10-C18**, respectively. A similar, but smaller, trend in the wettability of H_2_O and DMF on the fluorinated SAMs was also observed—however, due to the higher surface tension of the liquids, compared to ACN, a dramatic decrease in the contact angles of the **F8H10-C18** SAM with these liquids was not observed.

The wettability of nonpolar surfaces by nonpolar liquids arises from attractive dispersive interactions, which can reflect the chain packing density and surface morphology of the monolayers [25,95]. When nonpolar liquids were used (hexadecane and perfluorodecalin), the contact angles of the **F8H10-C10** and **F8H10-C12** SAMs were comparable, but lower than those obtained on the **F8H10SH** SAM, with the **F8H10-C18** SAM exhibiting the lowest values. The **F8H10-C10** and **F8H10-C12** SAMs displayed similar behavior toward the nonpolar contacting liquids, which corresponds to a predominant exposure of fluorocarbon chains at the interfaces. Nevertheless, the enhanced wettability of the **F8H10-C10** and **F8H10-C12** SAMs as compared to the **F8H10SH** SAM also suggests differences in chain density and orientation between the dithiol adsorbates and the **F8H10SH** SAM. A more tilted fluorocarbon chain and/or chain disorder can lead to an increase in atomic contact density per unit area on the interfaces, thereby contributing to the enhanced dispersive interactions between the probe liquids and surfaces [25]. The less densely packed structure, with its greater chain tilt and/or disorder in the fluorocarbon chains, is also expected to be more wettable than a more densely packed SAM. On the other hand, the interface of the **F8H10-C18** SAM is composed of a mixture of hydrocarbon and fluorocarbon moieties. The presence of the hydrocarbon moieties at the interface of the respective SAM should lead to a greater attractive dispersive interaction between hexadecane and the surface when compared to the former two SAMs.

Another important aspect to note from the contact angle data is the contact angle hysteresis (∆*θ* = *θ*_a_ − *θ*_r_) shown in Table 5, which can be used to indicate the surface roughness or the heterogeneity of an interface [85]. The hysteresis values of the bidentate **F8H10-Cm** SAMs are comparable to the values obtained for the corresponding monodentate SAMs. The calculated values suggest that the **F8H10-Cm** films examined here possess similar roughness/heterogeneity as their monothiol analogues. The contact angle measurements of the **F8H10-Cm** series suggest a mixed interface composed of both phase incompatible groups, proving to be a unique new class of adsorbate in the pursuit of homogeneously mixed dual-component monolayers on metal substrates [51].

## 3. Materials and Methods

### 3.1. Materials

The solvents hexanes, diethyl ether (Et_2_O), glacial acetic acid (AcOH), (all from Avantor Performance Materials, Center Valley, PA, USA), dimethyl sulfoxide (DMSO) and acetone (both from Fisher Scientific, Fair Long, NJ, USA) were used as received. Ethanol (EtOH, AAPER Alcohol and Chemical Co., Brookfield, CT, USA) was degassed by purging with nitrogen gas. Tetrahydrofuran (THF) was dried by distilling over calcium hydride (Sigma Aldrich, St. Louis, MO, USA). 7-Octen-1-ol (TCI America, Portland, OR, USA), 1-iodperfluorooctane (Synquest Lab, Alachua, FL, USA), triethylamine (Et_3_N), methanesulfonyl chloride (MsCl), azobisisobutyronitrile (AIBN), diethyl malonate, and 1-bromooctane (all from Sigma Aldrich) were used as received, unless otherwise noted. Zinc dust (Mallinckrodt, St. Louis, MO, USA), potassium iodide (KI, Acros, Fair Long, NJ, USA), Celite, sodium hydride (NaH), lithium aluminum hydride (LiAlH_4_) (all from Sigma Aldrich), sodium bicarbonate (NaHCO_3_, Arm & Hammer, Ewing, NJ, USA), magnesium sulfate (MgSO_4_, EDM Millipore Corporation, Billerica, MA, USA), hydrochloric acid (HCl) and sulfuric acid (H_2_SO_4_) (both from Avantor Performance Materials) were used as received unless otherwise noted. The silica gel used for column chromatography was purchased from Sorbent Technologies (Norcross, GA, USA). The silica gel TLC plates used during purification were purchased from Avantor Performance Materials. Nuclear magnetic resonance (NMR) spectra were obtained with CDCl_3_ (Cambridge Isotope Laboratories, Andover, MA, USA) as the solvent and used to collect the ^1^H NMR, ^13^C NMR, and ^19^F NMR spectra.

**Scheme 1:**
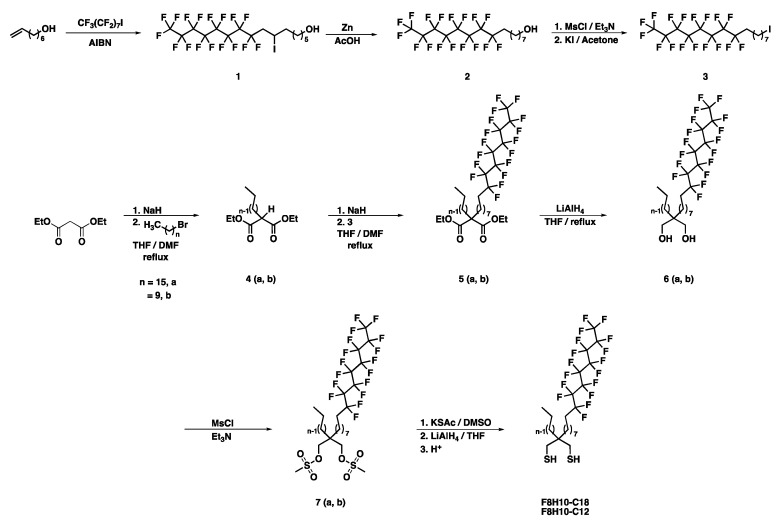
Strategy Used to Prepare 2-(9,9,10,10,11,11,12,12,13,13,14,14,15,15,16,16,16-Heptadecafluoro-hexadecyl)-2-hexadecylpropane-1,3-dithiol and 2-Decyl-2-(9,9,10,10,11,11,12,12,13,13,14,14,15,15,16,16,16-heptadecafluorohexadecyl)propane-1,3-dithi-ol (**F8H10-C18** and **F8H10-C12**, Respectively).

Gold shot (99.99%) was purchased from Kamis Inc., (Mahopac Falls, NY, USA). Chromium rods (99.9%) were purchased from R. D. Mathis Company (Long Beach, CA, USA). Polished single-crystal Si(100) wafers were purchased from Silicon Sense, Inc. (Nashua, NH, USA) and rinsed with absolute ethanol (EtOH, 200 proof, AAPER Alcohol and Chemical Co., Brookfield, CT, USA) before use. The contacting liquids used for wettability measurements were of the highest purity available from several sources and were used without purification: Water (H_2_O, generated from a Milli-Q Water System with resistance of 18.2 MΩ), *N*,*N*-dimethylformamide (DMF), acetonitrile (ACN), hexadecane (HD) (all from Sigma Aldrich, St. Louis, MO, USA), and perfluorodecalin (PFD, Synquest Labs, Alachua, FL, USA). The normal alkanethiols used to generate SAMs; *n*-decanethiol (**C10SH**) *n*-dodecanethiol (**C12SH**), and *n*-octadecanethiol (**C18SH**), were purchased from Aldrich Chemical Co. (St. Louis, MO, USA) and used without purification. *N,N*-Dimethylformamide (DMF), used as a solvent for organic synthetic reactions, was purchased from Aldrich Chemical Co., distilled over calcium hydride, and then stored under argon until use. We used established methods from the literature to synthesize the partially fluorinated adsorbates 11,11,12,12,13,13,14,14,15,15,16,16,17,17,18,18,18-heptadecafluorooctadecane-1-thiol (**F8H10SH**) [96] and 2-(9,9,10,10,11,11,12,12,13,13,14,14,15,15,16,16,16-heptadecafluorohexa-decyl)-2-octylpropane-1,3-dithiol (**F8H10-C10**) [62]. Similarly, we used the strategies shown in Scheme 1 to prepare 2-decyl-2-(9,9,10,10,11,11,12,12,13,13,14,14,15,15,16,16,16-heptadecafluorohexa-decyl)propane-1,3-dithiol (**F8H10-C12**) and 2-(9,9,10,10,11,11,12,12,13,13,14,14,15,15,16,16,16-hepta-decafluorohexadecyl)-2-hexadecylpropane-1,3-dithiol (**F8H10-C18**).

### 3.2. Synthesis of the Unsymmetrical Partially Fluorinated Spiroalkanedithiols

*9,9,10,10,11,11,12,12,13,13,14,14,15,15,15-Pentadecafluoro-7-iodopentadecan-1-ol* (**1**). In a dry 50-mL Schlenk flask, 7-octen-1-ol (0.25 g; 1.9 mmol), AIBN (0.033 g; 0.200 mmol), and 1-iodoperfluorooctane (1.27 g; 2.34 mmol) were mixed. The system was degassed using three cycles of a standard freeze-pump-thaw procedure. The reaction was then heated to 85 °C overnight. The reaction was cooled to rt to give **1** in 84% yield. The crude product was used directly in the next step without purification. ^1^H-NMR (400 MHz, CDCl_3_): δ 4.32 (m, 1H), 3.65 (t, *J* = 6.41 Hz, 2H), 2.75–2.94 (m, 2H), 1.71–1.85 (m, 2H), 1.56–1.60 (m, 2H), 1.24–1.41(m, 6H).

*9,9,10,10,11,11,12,12,13,13,14,14,15,15,16,16,16-Heptadecafluorohexadecan-1-ol* (**2**). In a 250 mL round bottom flask, 70 mL of AcOH was added to **1** (0.895 g; 1.33 mmol) and stirred until completely dissolved. Once dissolved, zinc dust (1.50 g; 23.0 mmol) was added to the flask, and allowed to stir at rt under a nitrogen environment. The reaction mixture was stirred for 48 h. Upon completion, the reaction was diluted with 200 mL of Et_2_O and then filtered through a bed of Celite. The Celite pad was washed with an additional 200 mL of Et_2_O. The organic phase was washed with water (10 × 100 mL) followed by a saturated NaHCO_3_ solution (10 g × 100 mL). The filtrate was washed with brine (2 × 100 mL), dried over MgSO_4_, filtered, and evaporated to dryness. The reaction afforded **2** in 74% yield. The crude product was used in the next step without further purification. ^1^H-NMR (400 MHz, CDCl_3_): δ 3.65 (t, *J* = 6.58 Hz, 2H), 1.99–2.09 (m. 2H), 1.55–1.60 (m, 4H), 1.24–1.41(m, 8H).

*1,1,1,2,2,3,3,4,4,5,5,6,6,7,7,8,8-Heptadecafluoro-16-iodohexadecane* (**3**). In a 250 mL round bottom flask, **2** (0.357 g; 0.651 mmol) was dissolved in anhydrous THF (50 mL) at 0 °C and stirred under a flow of nitrogen. An aliquot of Et_3_N (0.267 mL; 1.95 mmol) was added dropwise to the solution and allowed to stir for 20 min. Afterward, MsCl (0.20 mL; 2.60 mmol) was added dropwise. The reaction was allowed to stir at rt for 6 h under the flow of nitrogen. The reaction was then quenched with 50 mL of ice-cold water and extracted with Et_2_O (3 × 100 mL). The combined organic layers were washed with 1M HCl (1 × 100 mL), water (2 × 100 mL), and brine (2 × 100 mL), then dried over MgSO_4_, filtered, and evaporated to dryness. The crude mesylate was used in the next step without purification. In a 250 mL round bottom flask equipped with a condenser, the crude mesylate and KI (0.820 g; 4.94 mmol) were dissolved in acetone (100 mL). The solution was refluxed overnight at 60 °C. After cooling to rt, the solvent was removed with a rotary evaporator. The crude product was purified by column chromatography on silica gel (hexanes) to afford **3** in 75% yield. ^1^H-NMR (400 MHz, CDCl_3_): δ 3.21 (t, *J* = 6.87 Hz, 2H), 2.03–2.08 (m, 2H), 1.57–1.62 (m, 2H), 1.24–1.41(m, 10H).

*Diethyl 2-hexadecylmalonate* (**4a**). In a solution of THF (100 mL) and DMF (30 mL) at 0 °C was added sodium hydride (2.4 g; 60 mmol) under argon. Diethyl malonate (9.61 g, 60.0 mmol) was added slowly via an addition funnel. The mixture was stirred at rt for 15 min, followed by the addition of 1-bromohexadecane (6.8 g; 20 mmol). The mixture was heated under reflux for 2 h, cooled to rt, and concentrated under vacuum. The resultant oil was suspended in 100 mL of water. The mixture was extracted with hexanes (2 × 100 mL) and a 1:1 mixture of hexanes/diethyl ether (1 × 100 mL). The organic layers were combined and washed with water (3 × 50 mL) and brine (1 × 100 mL), dried over anhydrous magnesium sulfate, and evaporated to dryness. The crude product was purified by column chromatography on silica gel (hexanes/diethyl ether, 20:1 to 10:1) to afford **4a** in 89% yield. ^1^H-NMR (400 MHz, CDCl_3_): δ 4.19 (q, *J* = 7.2 Hz, 4H), 3.30 (t, *J* = 7.6 Hz, 1H), 1.88 (m, 2H), 1.36–1.20 (m, 34H), 0.87 (t, *J* = 6.6 Hz, 3H).

*Diethyl 2-decylmalonate* (**4b**) was prepared following an analogous procedure used to prepare **4a**. ^1^H-NMR (300 MHz, CDCl_3_): δ 4.19 (q, *J* = 6.9 Hz, 4H), 3.30 (t, *J* = 7.5 Hz, 1H), 1.87 (m, 2H), 1.36–1.20 (m, 22H), 0.87 (t, *J* = 6.6 Hz, 3H).

*Diethyl 2-(9,9,10,10,11,11,12,12,13,13,14,14,15,15,16,16,16-heptadecafluorohexadecyl)-2-hexadecyl-malonate* (**5a**). Sodium hydride (0.24 g; 6.0 mmol) was placed in a dry argon-flushed flask in a mixture of THF (50 mL) and DMF (20 mL) at 0 °C under an atmosphere of argon. To this solution, diester **4a** (0.75 g; 1.9 mmol) dissolved in THF (10 mL) was added dropwise via an addition funnel. The mixture was stirred at rt for 15 min, and a solution of **3** (1.7 g; 2.6 mmol) dissolved in THF (15 mL) was slowly added into the mixture. The reaction mixture was refluxed overnight, and then concentrated under vacuum. The resultant oil was suspended in water (100 mL), extracted with hexanes (2 × 50 mL), and followed by a 1:1 mixture of hexanes and diethyl ether (1 × 50 mL). The organic layers were washed with water (1 × 100 mL) and brine (1 × 100 mL), dried over anhydrous magnesium sulfate, and evaporated to dryness. The crude product was purified using column chromatography on silica gel (hexanes/diethyl ether, 20:1 to 10:1) to afford the pure compound **5a** in 49% yield. ^1^H-NMR (400 MHz, CDCl_3_): δ 4.17 (q, *J* = 7.2 Hz, 4H), 2.11–1.97 (m, 2H), 1.85 (m, 4H), 1.59 (m, 2H), 1.36–1.17 (m, 44H), 0.87 (t, *J* = 6.9 Hz, 3H).

*Diethyl 2-decyl-2-(9,9,10,10,11,11,12,12,13,13,14,14,15,15,16,16,16-heptadecafluorohexadecyl)malo-nate* (**5b**) was prepared following an analogous procedure used to prepare **5a**. ^1^H-NMR (300 MHz, CDCl_3_): δ 4.17 (q, *J* = 7.2 Hz, 4H), 2.15–1.93 (m, 2H), 1.85 (m, 4H), 1.58 (m, 2H), 1.36–1.10 (m, 32H), 0.87 (t, *J* = 6.6 Hz, 3H).

*2-(9,9,10,10,11,11,12,12,13,13,14,14,15,15,16,16,16-Heptadecafluorohexadecyl)-2-hexadecyl-propane-1,3-diol* (**6a**). A solution of diester **5a** (0.6 g; 0.7 mmol) in THF (100 mL) was added slowly to a slurry of lithium aluminum hydride (0.11 g; 2.9 mmol) in THF (10 mL) at 0 °C. The reaction mixture was refluxed for 3 h under an argon atmosphere. Afterward, the reaction was quenched with ethanol (25 mL), and acidified to a pH of ~1 by careful addition of a 1.0 M HCl solution (150 mL). The mixture was extracted with diethyl ether (3 × 100 mL). The organic layers were washed with a dilute HCl solution (1 × 100 mL), followed by brine (1 × 100 mL). The organic phase was then dried over anhydrous magnesium sulfate and filtered. The solvent was removed under vacuum, and the product purified by column chromatography on silica gel (hexanes/diethyl ether, 2:1) to afford diol **6a** in 84% yield. ^1^H-NMR (400 MHz, CDCl_3_): δ 3.57 (s, 4H), 2.11–1.97 (m, 2H), 1.59 (m, 2H), 1.42–1.20 (m, 42H), 0.87 (t, *J* = 6.9 Hz, 3H).

*2-Decyl-2-(9,9,10,10,11,11,12,12,13,13,14,14,15,15,16,16,16-heptadecafluorohexadecyl)pro-pane-1,3-diol* (**6b**) was prepared following an analogous procedure used to prepare **6a**. ^1^H-NMR (300 MHz, CDCl_3_): δ 3.57 (s, 4H), 2.15–1.93 (m, 2H), 1.59 (m, 2H), 1.42–1.20 (m, 30H), 0.87 (t, *J* = 7.2 Hz, 3H).

*2-(9,9,10,10,11,11,12,12,13,13,14,14,15,15,16,16,16-Heptadecafluorohexadecyl)-2-hexadecyl-propane-1,3-diyl dimethanesulfonate* (**7a**). A solution of diol **6a** (0.56 g; 0.67 mmol) and triethylamine (0.56 g; 4.8 mmol) in THF was prepared under the flow of argon. Methanesulfonyl chloride (0.48 g; 4.7 mmol) was added dropwise over 5 min to the solution. After the addition was completed, stirring was continued for 3 h at rt. Ice cold water (200 mL) was poured into the reaction mixture to destroy any excess methanesulfonyl chloride. The mixture was extracted with diethyl ether (3 × 100 mL). The organic phase was washed with a dilute HCl solution (1 × 100 mL), water (1 × 100 mL), and brine (1 × 100 mL). The organic layer was dried over anhydrous magnesium sulfate, and the solvent was removed by rotary evaporation to give the crude mesylate **7a** in 85% yield. The crude product was used directly in the next step without any purification. ^1^H-NMR (400 MHz, CDCl_3_): δ 4.03 (s, 4H), 3.03 (s, 6H), 2.11–1.98 (m, 2H), 1.58 (m, 2H), 1.42–1.17 (m, 42H), 0.87 (t, *J* = 6.6 Hz, 3H).

*2-Decyl-2-(9,9,10,10,11,11,12,12,13,13,14,14,15,15,16,16,16-heptadecafluorohexadecyl)pro-pane-1,3-diyl dimethanesulfonate* (**7b**) was prepared following an analogous procedure used to prepare **7a**. ^1^H-NMR (300 MHz, CDCl_3_): δ 4.03 (s, 4H), 3.03 (s, 6H), 2.15–1.93 (m, 2H), 1.58 (m, 2H), 1.42–1.17 (m, 30H), 0.88 (t, *J* = 6.9 Hz, 3H).

*2-(9,9,10,10,11,11,12,12,13,13,14,14,15,15,16,16,16-Heptadecafluorohexadecyl)-2-hexadecyl-propane-1,3-dithiol* (**F8H10-C18**). Dimesylate **7a** (0.4 g; 0.4 mmol) was dissolved in anhydrous DMSO (70 mL) and added to a reaction flask containing potassium thioacetate (0.25; 2.2 mmol) suspended in DMSO (10 mL). The reaction was heated to 120 °C for 24 h under an atmosphere of argon. After the reaction was cooled to rt, water (50 mL) and brine (50 mL) were poured into the solution, and the mixture was extracted with diethyl ether (3 × 100 mL). The organic phases were combined and washed with water (3 × 100 mL) and brine (1 × 100 mL), dried over magnesium sulfate, filtered, and evaporated to dryness. The dithioacetate dissolved in THF (80 mL) was added into a slurry of lithium aluminum hydride (0.17 g; 4.5 mmol) in THF (10 mL) slowly at 0 °C. The reaction mixture was then refluxed for 3 h under an atmosphere of argon and then quenched with ethanol (25 mL, previously degassed). The mixture was acidified to pH ~1 by careful addition of 1.0 M H_2_SO_4_ (previously degassed) and then extracted with diethyl ether (3 × 100 mL). The organic layers were combined and washed with a dilute HCl solution (1 × 100 mL) and brine (1 × 100 mL). The organic phase was dried over anhydrous magnesium sulfate and filtered. After removal of the solvent under vacuum, the impurities were removed by column chromatography on silica gel (hexanes), affording a mixture of dithiol and the corresponding disulfide. The reduction and purification were repeated to afford pure dithiol **8a** in 54% yield. However, the product still contained ~3% of the corresponding disulfide. ^1^H NMR (600 MHz, CDCl_3_): δ 2.87 (s, disulfide contaminant, ~3%), 2.51 (d, *J* = 8.25 Hz, 4H), 2.15–1.98 (broad m, 2H), 1.58 (m, 2H), 1.42–1.17 (m, 42H), 1.07 (t, *J* = 8.60 Hz, 2H), 0.87 (t, *J* = 6.87 Hz, 3H). ^13^C-NMR (150 MHz, CDCl_3_): δ 39.8 (s), 36.6 (s), 33.7 (s), 32.0 (s), 30.8 (t, *J* = 22.2 Hz), 30.3–29.1 (m), 23.2 (s), 22.7 (s), 20.1 (s) 14.1 (s). Broad peaks at δ 120.4–106.6 are characteristic of a long perfluorocarbon chain [73]. ^19^F-NMR (565 MHz, CDCl_3_): δ −80.6 (m, 3F), −114.3 (m, 2F), −121.7 (m, 6F), −122.6 (m, 2F), −123.4 (m, 2F), −126.1 (m, 2F). CI-MS, *m*/*z*: 862.3367 [M^+^], 861.3340 [M^+^–H], 860.3312 [M^+^ − 2H]. 

*2-Decyl-2-(9,9,10,10,11,11,12,12,13,13,14,14,15,15,16,16,16-heptadecafluorohexadecyl)propane-1,3-dithiol* (**F8H10-C12**) was prepared following an analogous procedure used to prepare **F8H10-C12**. ^1^H NMR (600 MHz, CDCl_3_): δ 2.87 (s, disulfide contaminant, ~10%), 2.52 (d, *J* = 9.0 Hz, 4H), 2.15–1.98 (broad m, 2H), 1.58 (broad, 2H), 1.42–1.17 (m, 30H), 1.09 (t, *J* = 8.4 Hz, 2H), 0.87 (t, *J* = 6.9 Hz, 3H). ^13^C NMR (150 MHz, CDCl_3_): δ 39.8 (s), 36.8 (s), 33.6 (s), 31.9 (s), 30.8 (t, *J* = 22.18 Hz), 30.3–29.1 (m), 23.2 (s), 22.7(s), 20.1(s) 14.1 (s). Broad peaks at δ 120.4–106.6 are characteristic of a long perfluorocarbon chain [73]. ^19^F -NMR (565 MHz, CDCl_3_): δ −80.8 (m, 3F), −114.4 (m, 2F), −121.7 (m, 6F), −122.7 (m, 2F), −123.5 (m, 2F), −126.1 (m, 2F). CI-MS, *m*/*z*: 778.2389 [M^+^], 777.2396 [M^+^–H], 776.2366 [M^+^–2H].

### 3.3. Characterization Techniques

#### 3.3.1. Preparation of the Substrates

Gold substrates were prepared by thermal evaporation under vacuum (1 × 10^−5^ Torr) of a 100 Å adhesive layer of chromium onto the surface of polished silicon wafers (previously rinsed with absolute ethanol and blown dried with a stream of ultra-pure nitrogen) followed by the evaporation of 1000 Å of gold at an evaporation rate of 1 Å/s for both metals. The gold-coated wafers were cut with a diamond-tipped stylus into slides (1 × 4 cm), rinsed with absolute ethanol, and dried with a stream of ultra-pure nitrogen before collecting base ellipsometric constants and immersing in the thiol solutions.

#### 3.3.2. Preparation of SAMs

All vials used to form SAMs were previously cleaned with piranha solution (7:3 H_2_SO_4_:H_2_O_2_). *Caution: Piranha solution reacts violently with organic materials and should be handled carefully!* Solutions of 1 mM of each adsorbate were prepared in the indicated solvents: Normal alkanethiols in ethanol, **F8H10SH** in ethanol, and the **F8H10-Cm** adsorbates in DMF. The freshly evaporated gold substrates were then incubated in each thiol solution for a period of 48 h at rt. Prior studies have noted that the chelating dithiol adsorbates need longer adsorption/equilibration time (i.e., 48 h) than normal alkanethiols (24 h) to generate SAMs to have maximum coverage [61,67]. Prior to instrumental analysis, the slides were rinsed with THF (only the fluorinated adsorbates), toluene, ethanol, and blown dry with ultra-pure nitrogen.

#### 3.3.3. Ellipsometry

Thickness measurements were performed on a Rudolph Research Auto EL III ellipsometer which was equipped with a He-Ne laser at a wavelength of 632.8 nm. The sample stage was set to correspond with an angle of incidence of 70°. A refractive index of 1.45 was used throughout the thickness measurements for all films. The reported data are an average collected from two separate slides with three different spots per slide.

#### 3.3.4. Contact Angle Goniometry

A Ramé-Hart model 100 contact angle goniometer was employed to measure the contact angle of the SAMs. The contacting liquids, water (H_2_O), hexadecane (HD), perfluorodecalin (PFD), *N,N*-dimethylformamide (DMF), and acetonitrile (ACN) were dispensed (advancing angle, *θ*_a_) and withdrawn (receding angle, *θ*_r_) on the surfaces of the SAMs using a Matrix Technologies micro-Electrapette 25 at the slowest possible speed (1 μL/s). The measurements were performed at rt with the pipet tip in contact with the drop. Reported contact angles for each sample are an average of 12 measurements. Measurements were taken from two separate slides with three drops per slide using both drop edges. 

#### 3.3.5. PM-IRRAS

Polarization modulation infrared reflection absorption spectroscopy (PM-IRRAS) data were collected using a Nicolet NEXUS-IR 670 Fourier transform spectrometer equipped with a liquid nitrogen-cooled mercury-cadmium-telluride (MCT) detector and a Hinds Instruments PEM-90 photoelastic modulator. The *p*-polarized light was incident at 80°. The spectra were collected using 512 scans for the C-H stretching region (2700–3100 cm^−1^) and 1024 scans for the C-F stretching region (1000–1400 cm^−1^) with a spectral resolution of 4 cm^−1^.

#### 3.3.6. XPS

X-ray photoelectron spectroscopy (XPS) spectra were collected using a PHI 5700 X-ray photoelectron spectrometer equipped with a monochromatic Al Kα X-ray source (hν = 1486.7 eV) incident at 90° relative to the axis of a hemispherical energy analyzer. The spectrometer was operated at high resolution with a pass energy of 23.5 eV, a photoelectron takeoff angle of 45° from the surface, and an analyzer spot diameter of 1.1 mm. The base pressure in the chamber during measurements was ~2 × 10^−8^ Torr, and the spectra were collected at rt. Ten scans were used to obtain the C 1s and F 1s spectra, while five and thirty scans were used to obtain the Au 4f and S 2p spectra, respectively. After collecting the data, the binding energies were referenced by setting the Au 4f_7/2_ binding energy to 84.0 eV. The peak intensities were quantified by standard curve-fitting software using Shirley background subtraction and Gaussian-Lorentzian profiles.

## 4. Conclusions

We have examined SAMs generated from a series of unsymmetrical partially fluorinated spiroalkanedithiols having tailgroups comprised of a terminally fluorinated chain and a hydrocarbon chain of varying lengths, **F8H10-Cm** where m = 10, 12, and 18, and compared them to the corresponding normal and fluorinated alkanethiols. Analysis from XPS reveal the **F8H10-Cm** adsorbates bind to the gold substrate via both sulfur headgroups. The films revealed interfacial properties corresponding to both the hydrocarbon and fluorocarbon chains incorporated into the adsorbates. The branched nature of these adsorbates was found to influence the resulting films in which moderately ordered, loosely packed SAMs were formed when compared to the single-chained analogs. When considering the influence of the hydrocarbon chain length in the double-chained structure, the impact of the addition of two methylene units appears to be negligible. In contrast, increasing the hydrocarbon chain length to be identical with the partially fluorinated alkyl chain in the **F8H10-C18** adsorbate was found to generate SAMs with a greater degree of loose packing and chain disorder, which can be attributed, at least in part, to phase-incompatible fluorocarbon-hydrocarbon interactions. Finally, the monolayers obtained from the **F8H10-Cm** adsorbates showed no large-scale roughness or heterogeneity, demonstrating the potential for the creation of homogeneously mixed dual-component SAMs from these types of adsorbates. 

## Figures and Tables

**Table 1:** Selected studies of partially fluorinated self-assembled monolayers (SAMs) in the literature.

Adsorbate	Effect on Wettability	Reference
F_3_C(CH_2_)_n_SH n = 12–15	Oriented “FC-HC” dipoles at the monolayer terminus lead to fluorocarbon SAMs that are more wettable by polar contacting liquids than hydrocarbon SAMs.	[45,46,47]
F_3_C(CF_2_)_n_(CH_2_)_m_SH n = 0–9; m = 15–6	Progressively fluorinated hexadecanethiols where the “FC-HC” dipole is buried into the film reveal a diminished effect on wettability with increasing fluorination.	[46]
F_3_C(CF_2_)_n_(CH_2_)_m_SH n = 0–9; m = 11	Dispersive surface energies decrease as the amount of fluorination increases until it reaches a plateau for n = 5–9, due to a decrease in the surface density of CF_3_ groups.	[39,48]

**Table 2:** Ellipsometric thickness measurements for SAMs on gold derived from the monodentate thiols and the **F8H10-Cm** dithiols.

Adsorbate	Thickness (Å)
**C10SH**	10 ± 2 Å
**C12SH**	12 ± 2 Å
**C18SH**	22 ± 2 Å
**F8H10SH**	18 ± 2 Å
**F8H10-C10**	14 ± 2 Å
**F8H10-C12**	16 ± 2 Å
**F8H10-C18**	21 ± 2 Å

**Table 3:** XPS peak positions for the C 1s and F 1s regions for the partially fluorinated SAMs.

Adsorbate	Peak Position (eV)			
	C 1s (CH_2_)	C 1s (CF_2_)	C 1s (CF_3_)	F 1s
**C10SH**	284.7	-	-	-
**C12SH**	284.8	-	-	-
**C18SH**	285.3	-	-	-
**F8H10SH**	284.7	291.2	293.5	688.4
**F8H10-C10**	284.7	291.6	293.9	688.9
**F8H10-C12**	284.5	291.4	293.6	688.7
**F8H10-C18**	284.4	291.4	293.6	688.7

**Table 4:** XPS Intensities for C 1s and F 1s regions of each fluorinated SAM.

Adsorbate	C 1s ^a^ (CH_2_)	C 1s ^a^ (CF_2_ + CF_3_)	F 1s ^b^
**F8H10SH**	0.96	1.00	1.00
**F8H10-C10**	1.16	0.72	0.72
**F8H10-C12**	1.07	0.71	0.69
**F8H10-C18**	1.50	0.57	0.55

^a^ Peak intensities were normalized with respect to the intensity of the fluorocarbon components (i.e., CF_2_ + CF_3_) in the C 1s region of the **F8H10SH** SAM; ^b^ Peak intensities were normalized with respect to the intensity of the F 1s region of the **F8H10SH** SAM.

**Table 5:** Advancing contact angles (*θ*_a_, °) on the indicated SAMs using various probe liquids with hysteresis values (∆*θ* = *θ*_a_ − *θ*_r_) ^a^ given in parentheses.

	H_2_O	DMF	ACN	HD	PFD
**C10SH**	116 (7)	74 (9)	61 (11)	47 (6)	37 (8)
**C12SH**	116 (8)	75 (10)	62 (10)	48 (7)	38 (8)
**C18SH**	116 (9)	75 (9)	67 (10)	50 (7)	40 (9)
**F8H10SH**	127 (8)	87 (8)	83 (10)	80 (7)	47 (10)
**F8H10-C10**	127 (11)	87 (9)	82 (13)	75 (7)	37 (11)
**F8H10-C12**	125 (8)	87 (8)	82 (10)	73 (7)	33 (7)
**F8H10-C18**	121 (7)	81 (10)	68 (11)	59 (7)	26 (5)

^a^ Receding angles, *θ*_r_.

## Supplementary Materials

The following are available online, Figure S1: ^1^H NMR **F8H10-C12**, Figure S2: ^13^C NMR **F8H10-C12**, Figure S3: ^19^F NMR **F8H10-C12**, Figure S4: ^1^H NMR **F8H10-C18**, Figure S4: ^13^C NMR **F8H10-C18**, Figure S6: ^19^F NMR **F8H10-C18**, Table S1: Surface Tension, Polar, and Dispersive Components, Table S2: Calculated Surface Energy Values of the SAMs.
