# Supplementary material for: Unsymmetrical Spiroalkanedithiols Having Mixed Fluorinated and Alkyl Tailgroups of Varying Length: Film Structure and Interfacial Properties
Source: Molecules. 2018 Oct 13;23(10):2632. doi: 10.3390/molecules23102632 (PMC6222720; doi:10.3390/molecules23102632)
Supplement: Supplementary file 1 [file molecules-23-02632-s001.pdf]

Supplementary Materials

for

Unsymmetrical Spiroalkanedithiols Having Mixed Fluorinated and Alkyl Tailgroups of Varying Length: Film Structure and Interfacial Properties

*Pawilai Chinwangso, Lydia R. St. Hill, Maria D. Marquez, and T. Randall Lee**

Department of Chemistry and the Texas Center for Superconductivity

University of Houston, 4800 Calhoun Road, Houston, Texas 77204-5003, United States

*Corresponding author: trlee@uh.edu

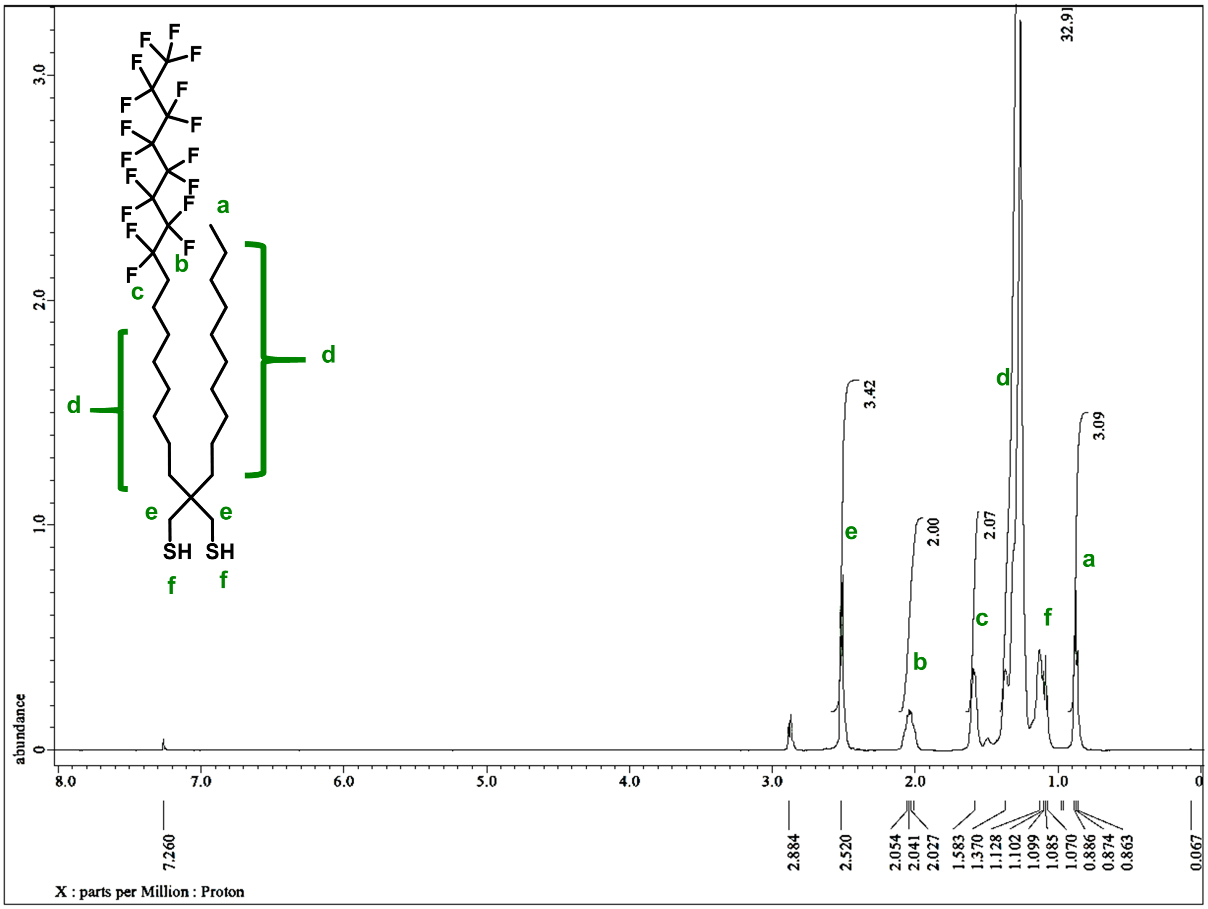

Figure S1. ^{1}H NMR of 2-decyl-2-(9,9,10,10,11,11,12,12,13,13,14,14,15,15,16,16,16-heptadeca-fluorohexadecyl)propane-1,3-dithiol (**F8H10-C12**).

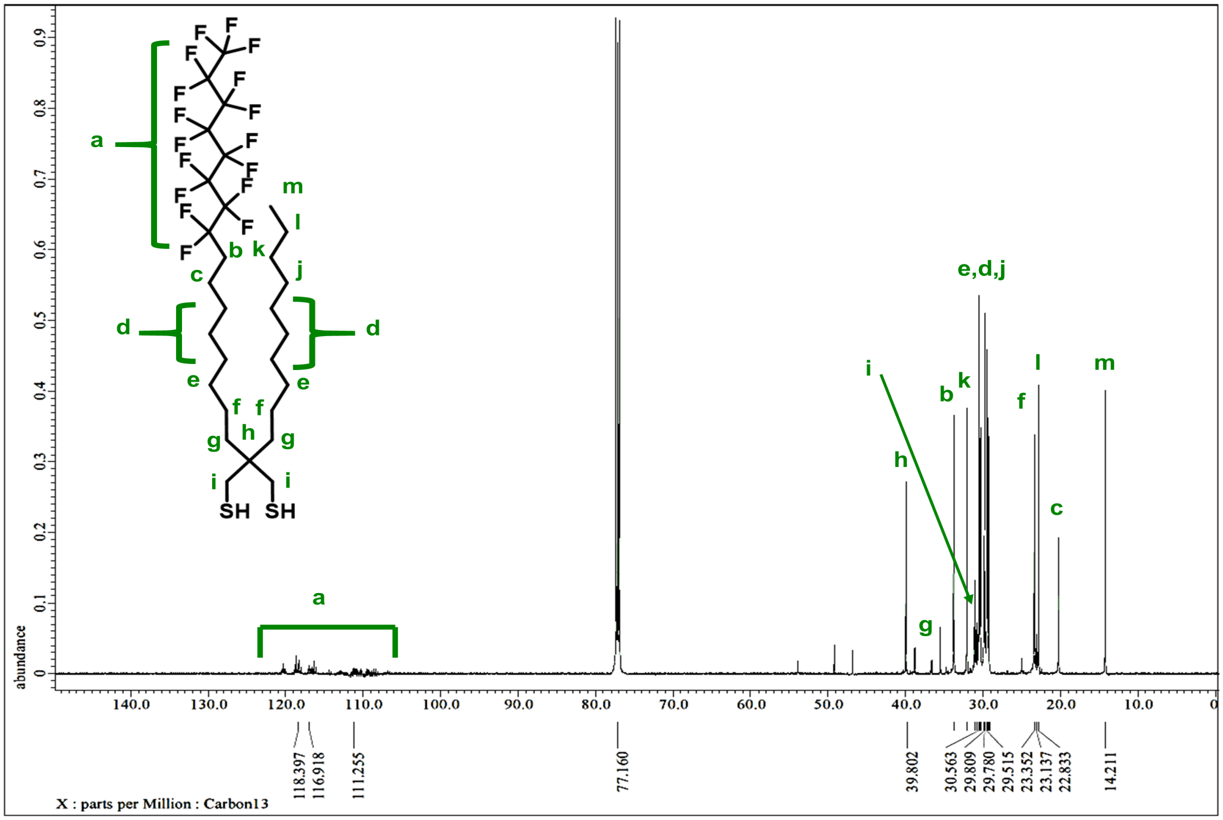

Figure S2. ^{13}C NMR of 2-decyl-2-(9,9,10,10,11,11,12,12,13,13,14,14,15,15,16,16,16-heptadeca-fluorohexadecyl)propane-1,3-dithiol (**F8H10-C12**).

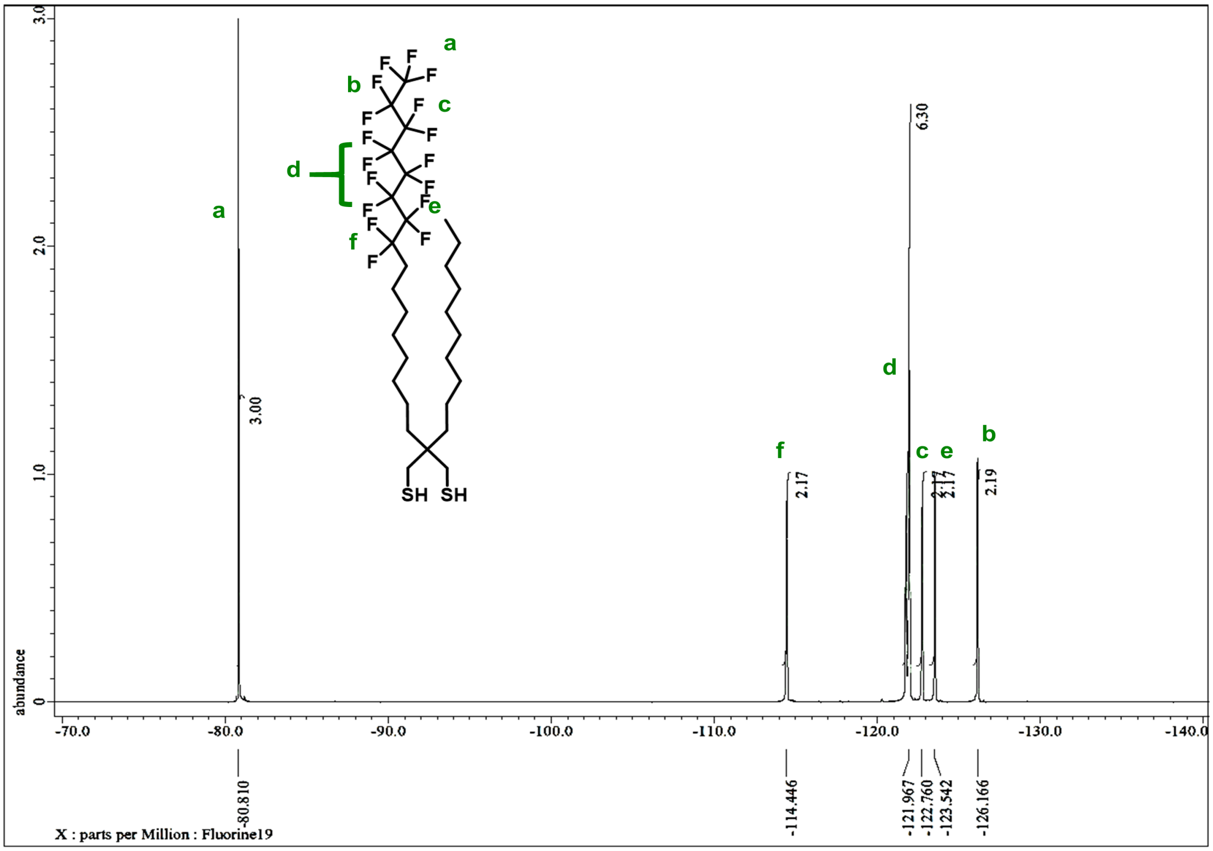

Figure S3. ^{19}F NMR of 2-decyl-2-(9,9,10,10,11,11,12,12,13,13,14,14,15,15,16,16,16-heptadeca-fluorohexadecyl)propane-1,3-dithiol (**F8H10-C12**).

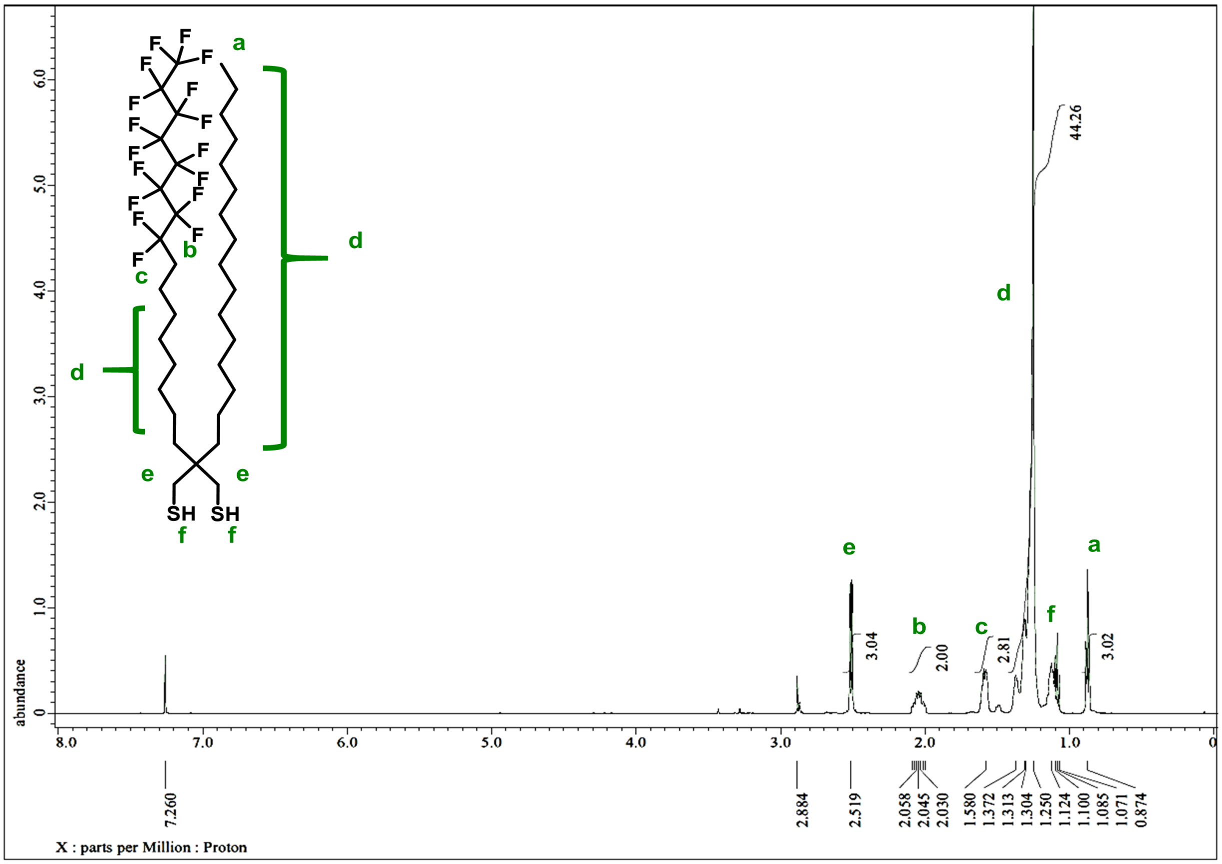

Figure S4. ^{1}H NMR of 2-(9,9,10,10,11,11,12,12,13,13,14,14,15,15,16,16,16-heptadeca-fluorohexadecyl)-2-hexadecylpropane-1,3-dithiol (**F8H10-C18**).

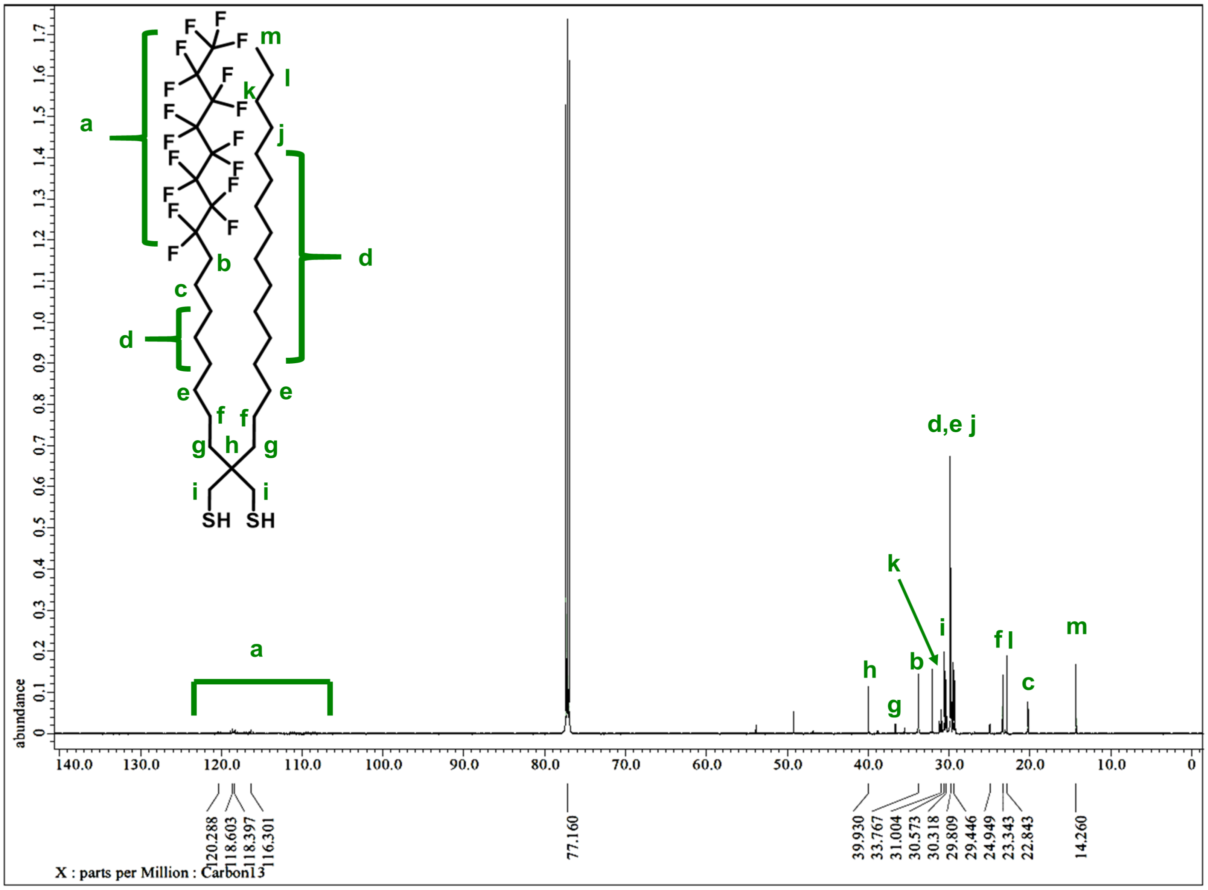

Figure S5. ^{13}C NMR of 2-(9,9,10,10,11,11,12,12,13,13,14,14,15,15,16,16,16-heptadeca-fluorohexadecyl)-2-hexadecylpropane-1,3-dithiol (**F8H10-C18**).

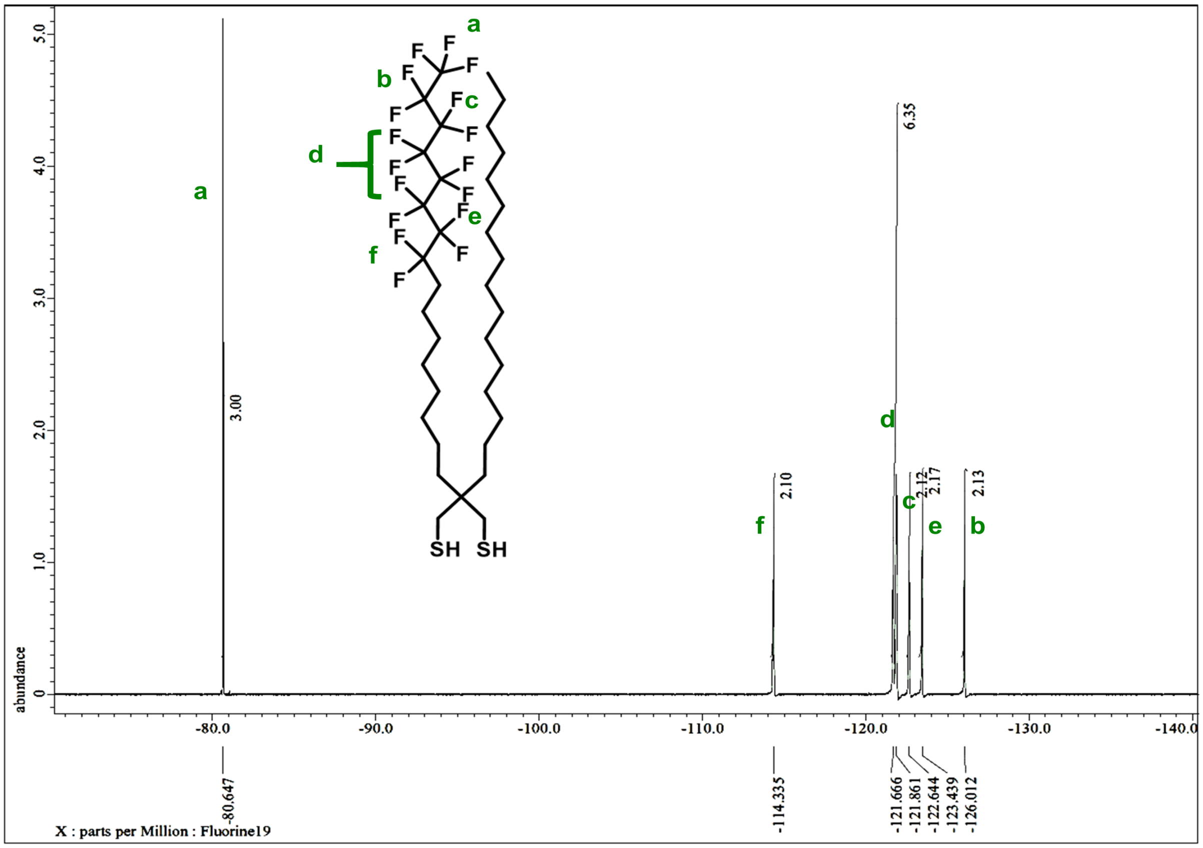

Figure S6. ^{19}F NMR of 2-(9,9,10,10,11,11,12,12,13,13,14,14,15,15,16,16,16-heptadeca-fluorohexadecyl)-2-hexadecylpropane-1,3-dithiol (**F8H10-C18**).

Surface Energy Calculations. The surface energy of the SAMs can be estimated using the contact angle values, found in Table 4, and Youngs' equation (1) [S1-S4],

$$\gamma_{SV} = \gamma_{LV}\cos\theta + \gamma_{SL} \quad (1)$$

Where γ_{SV} is the surface energy of the solid, γ_{LV} is the surface tension of the liquid, θ is the contact angle, and γ_{SL} is the interfacial energy of the solid/liquid. Using the Owens-Wendt method, γ_{SL} can be determined according to equation 2.

$$\gamma_{SL} = \gamma_{SV} + \gamma_{LV} - 2\left(\sqrt{\gamma_{SV}^{D}\gamma_{LV}^{D}} + \sqrt{\gamma_{SV}^{P}\gamma_{LV}^{P}}\right) \quad (2)$$

Where γ_{SV}^{D} and γ_{LV}^{D} are the dispersive components and γ_{SV}^{P} and γ_{LV}^{P} are the polar components. Substitution into equation 1, gives equation 3.

$$\sqrt{\gamma_{SV}^{D}\gamma_{LV}^{D}} + \sqrt{\gamma_{SV}^{P}\gamma_{LV}^{P}} = \frac{1}{2}\left[\gamma_{LV}(1 + \cos\theta)\right] \quad (3)$$

To use the Owens-Wendt method, the contact angle, the surface tension, as well as the polar and dispersive components of the surface tension of two contacting liquids must be known. For the SAMs in this study, the surface energies were estimated using the contact angles of H_2O and HD. The surface tension and the polar and dispersive components of the contacting liquids are listed in Table S1[S2-S3]. The surface energies of the SAMs, listed in Table S2, were calculated using equation 3 and the values found in Table S1.

Table S1. Surface Tension, Polar, and Dispersive Components for Water and Hexadecane

**Liquid**	γ_{LV} (mN·cm^{-1})	γ_{LV}^{P} (mN·cm^{-1})	γ_{LV}^{D} (mN·cm^{-1})
**Water**	72.1	52.2	19.9
**Hexadecane**	27.5	0	27.5

Table S2. Calculated Surface Energy Values of the SAMs

Adsorbate	Surface Energy (mJ/m^2)
**C10SH**	19.4
**C12SH**	19.1
**C18SH**	18.6
**F8H10SH**	9.5
**F8H10-C10**	10.9
**F8H10-C12**	11.5
**F8H10-C18**	15.8

References

(S1) Chen, J.; Wang, Z.; Oyola-Reynoso, S.; Thuo, M. M. Properties of Self-Assembled Monolayers Revealed via Inverse Tensiometry. *Langmuir* **2017**, *33*, 13451–13467. DOI: 10.1021/acs.langmuir.7b01937

(S2) Shon, Y.-S.; Lee, S.; Colorado, R.; Perry, S. S.; Lee, T. R. Spiroalkanedithiol-Based SAMs Reveal Unique Insight into the Wettabilities and Frictional Properties of Organic Thin Films. *J. Am. Chem. Soc.* **2000**, *122*, 7556–7563. DOI: 10.1021/ja000403z.

(S3) Colorado, R.; Lee, T. R. Wettabilities of Self-Assembled Monolayers on Gold Generated from Progressively Fluorinated Alkanethiols. *Langmuir* **2003**, *19*, 3288–3296. DOI: 10.1021/la0263763.

(S4) Takenaga, M.; Jo, S.; Graupe, M.; Lee, T. R. Effective van der Waals Surface Energy of Self-Assembled Monolayer Films having Systematically Varying Degrees of Molecular Fluorination. *J. Colloid Interface Sci.* **2008**, *320*, 264–267. DOI: 10.1016/j.jcis.2007.12.048.
